# Effects of Different Irrigation Protocols on the Surface Morphology and Elemental Composition of Super One File Nickel–Titanium Instruments

**DOI:** 10.3390/bioengineering13091061

**Published:** 2026-09-12

**Authors:** Tufan Ozasir, Birgul Ozasir, Gulsah Unsal, Kamran Gulsahi

**Affiliations:** Department of Endodontics, Faculty of Dentistry, Baskent University, Etimesgut, Ankara 06790, Turkey; beren@baskent.edu.tr (B.O.); gulsahunsal@baskent.edu.tr (G.U.); kgulsahi@baskent.edu.tr (K.G.)

**Keywords:** corrosion, elemental composition, HEDP, nickel–titanium instruments, sodium hypochlorite, surface morphology

## Abstract

**Background:** Nickel–titanium (Ni-Ti) instruments are continuously exposed to irrigating solutions during root canal preparation, which may affect their surface integrity. Evidence regarding the combined morphological and elemental effects of contemporary irrigation protocols on heat-treated single-file systems remains limited. This study evaluated the effects of different irrigation protocols on the surface morphology and elemental composition of Super One File Ni-Ti instruments using scanning electron microscopy (SEM) and energy-dispersive X-ray spectroscopy (EDS). **Methods:** Thirty Super One File instruments (size 25/.04) were randomly allocated to three groups: distilled water (DW), a sequential NaOCl–EDTA–NaOCl irrigation protocol (NEN), and a NaOCl/HEDP continuous chelation protocol. Instruments were continuously rotated in the assigned solution for 20 min. Surface morphology was assessed by SEM and elemental composition by EDS. SEM data were analysed using the Fisher–Freeman–Halton exact test, whereas EDS data were analysed using linear mixed-effects models with post hoc pairwise comparisons (α = 0.05). **Results:** SEM analysis revealed protocol- and region-dependent differences in surface alterations. The NEN group showed significantly higher corrosion and pitting frequencies than the DW group in selected regions, whereas no significant differences in these outcomes were detected between the NEN and HEDP groups. EDS analysis showed significant protocol × region interactions for all elements except Cl. These differences were most pronounced in the coronal region, where the HEDP group exhibited lower Ni and Ti and higher O, P, Na, and Ca levels than the DW and NEN groups. **Conclusions:** The tested irrigation protocols produced distinct, region-dependent surface responses in Super One File instruments, highlighting the potential influence of irrigation chemistry on Ni-Ti instrument surfaces under controlled experimental conditions. The clinical and mechanical implications of these alterations remain to be established.

## 1. Introduction

Effective root canal debridement relies on the combined action of mechanical instrumentation and chemical irrigation. Consequently, Ni-Ti instruments are exposed to irrigating solutions throughout canal preparation [1,2].

Nickel–titanium (Ni-Ti) instruments have revolutionized root canal preparation because of their superior flexibility, shape memory, and fatigue resistance, enabling safer and more efficient instrumentation of curved root canals than stainless steel instruments [3]. The clinical performance of contemporary Ni-Ti instruments depends not only on their design and metallurgical characteristics but also on the integrity of their surface. The corrosion resistance of Ni-Ti alloys is largely attributed to a stable titanium oxide layer; however, exposure to chemically active irrigants may compromise this protective layer, potentially altering the surface integrity and mechanical behaviour of the instruments [4,5].

The chemical environment to which Ni-Ti instruments are exposed is determined by the irrigation protocol. Sodium hypochlorite (NaOCl) is widely used for its tissue-dissolving and antimicrobial properties, whereas ethylenediaminetetraacetic acid (EDTA) is commonly used for chelation [6,7]. More recently, 1-hydroxyethylidene-1,1-diphosphonic acid (HEDP), which is compatible with NaOCl, has enabled continuous chelation during instrumentation [8,9]. These chemically distinct protocols may interact differently with Ni-Ti instrument surfaces, potentially resulting in corrosion, pitting, or other surface alterations [10].

Previous studies have demonstrated that exposure to irrigating solutions can affect both the surface morphology and elemental composition of Ni-Ti instruments using complementary surface-analysis techniques, including SEM, AFM, and EDS [5,11,12,13]. However, relatively few studies have simultaneously evaluated morphological and elemental surface changes in heat-treated Ni-Ti instruments [14,15,16,17]. Moreover, information regarding their response to sequential irrigation and HEDP-based continuous chelation remains limited, particularly with respect to region-dependent morphological and elemental changes.

Super One File^®^ (Perfect, Shenzhen Perfect Medical Instruments Co., Ltd., Shanwei City, China) is a recently introduced continuous-rotation single-file Ni-Ti system incorporating a proprietary double heat-treatment technology, as specified by the manufacturer. Thermomechanical processing can influence the corrosion resistance and surface behaviour of Ni-Ti alloys [4], and single-file systems may be exposed to irrigating solutions for a relatively longer cumulative duration than multi-file systems [18]. Therefore, the interaction of this system with different irrigation protocols warrants investigation. However, information regarding its morphological and elemental response to such protocols remains limited.

Accordingly, this study aimed to evaluate the effects of sequential NaOCl–EDTA–NaOCl irrigation and HEDP-based continuous chelation on the surface morphology and elemental composition of the apical, middle, and coronal regions of Super One File instruments using SEM and EDS, with distilled water serving as the control. The null hypothesis was that the tested irrigation protocols would not result in significant differences in surface morphology or elemental composition.

## 2. Materials and Methods

### 2.1. Sample Selection

The sample size was determined using an a priori power analysis performed with G*Power software (version 3.1.9.7, Heinrich Heine University, Düsseldorf, Germany), assuming a significance level of α = 0.05, a statistical power of 0.80, and an effect size of f = 0.68. The effect size (f = 0.68) was derived from the surface carbon-content data reported in the EDS analysis of Samara et al. [6]. The analysis indicated that a minimum of 10 samples per group were required. A total of 30 brand new, sterile, single-use, tip size 25/.04, 21 mm length nickel–titanium (Ni-Ti) single-file instruments (Super One File^®^, Perfect, Shenzhen Perfect Medical Instruments Co., Ltd., Shanwei City, China) were used in this study.

### 2.2. Preparation of Solutions

The solutions used in the study were distilled water, 5% sodium hypochlorite (NaOCl; Werax, Izmir, Turkey), and 17% ethylenediaminetetraacetic acid (EDTA; Werax, Izmir, Turkey). All solutions were used as received from the manufacturers without further dilution or chemical modification. The HEDP-based solution (Dual Rinse HEDP; Medcem GmbH, Weinfelden, Switzerland) was prepared immediately before use by mixing one capsule (0.9 g) of etidronic acid with 10 mL of 5% NaOCl for 2 min, in accordance with the manufacturer’s instructions.

### 2.3. Experimental Protocol

The instruments were randomly assigned into three groups (*n* = 10):

- Control group: Distilled water (DW) for 20 min.
- Sequential irrigation group (NEN): 5% NaOCl for 10 min, followed by 17% EDTA for 1 min and a final 5% NaOCl irrigation for 9 min.
- Continuous chelation group (HEDP): NaOCl/Dual Rinse^®^ HEDP mixture for 20 min.

**Figure 1:**
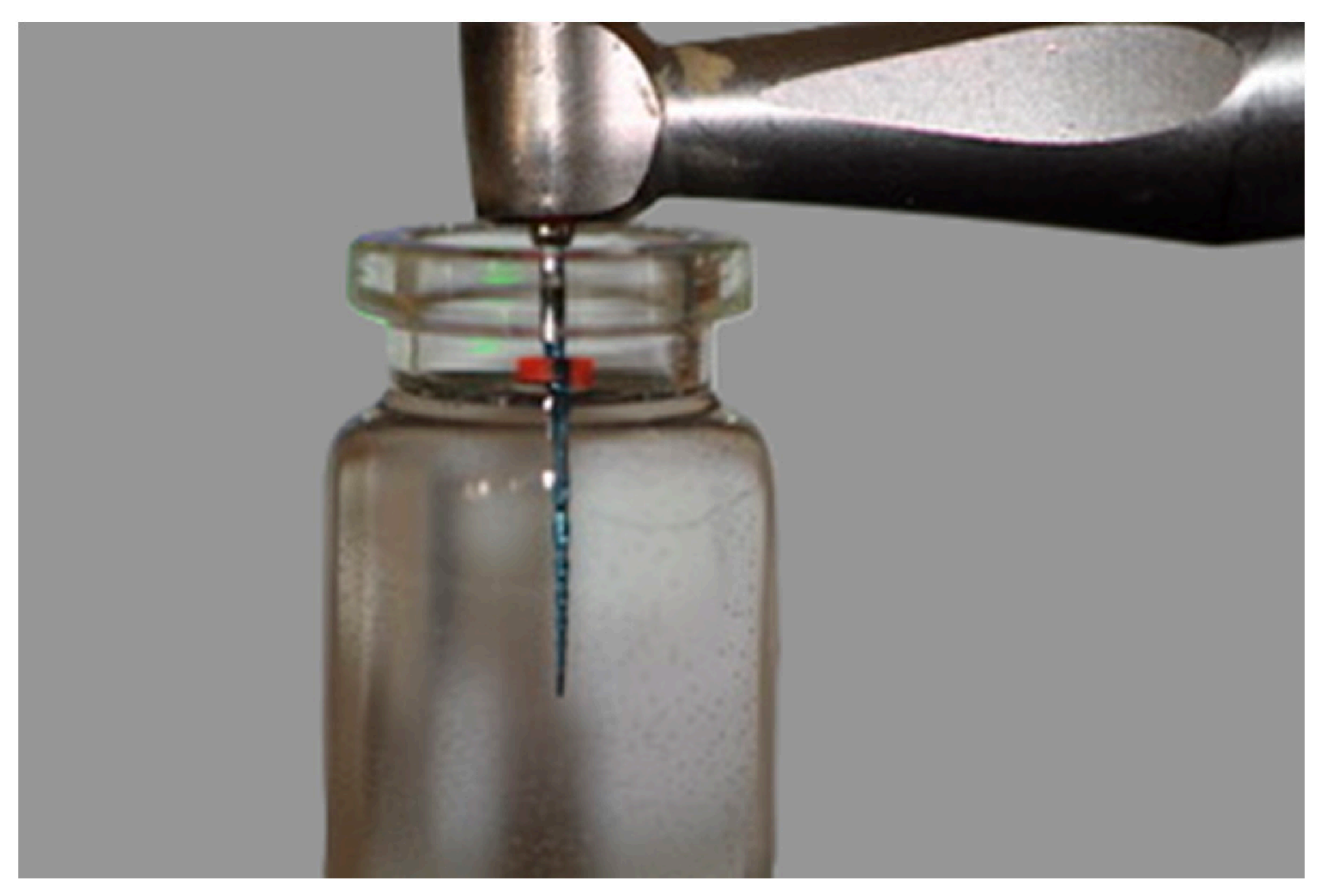
Experimental setup used for standardized immersion and continuous rotation of the Ni-Ti instruments in the assigned irrigation solutions.

In the NEN group, the instruments were rinsed with saline between solutions. The endodontic motor (VDW Silver; VDW, Munich, Germany) was fixed in place and not hand-held. For each instrument, a separate glass tube containing the assigned irrigating solution was used to prevent cross-contamination. Only the active 16 mm tip portion of each instrument was immersed in the assigned irrigating solution while being continuously rotated according to the manufacturer’s recommended settings. The instruments were operated at 400 rpm and 2.5 N·cm torque for 20 min in their respective irrigation solutions throughout the experimental period (Figure 1). All experimental procedures were performed at room temperature.

Following the experimental procedure, all instruments were rinsed with saline solution to remove residual irrigants and gently dried with sterile gauze. The samples were subsequently individually packaged and stored under sterile conditions until further analysis.

### 2.4. SEM Analysis

**Figure 2:**
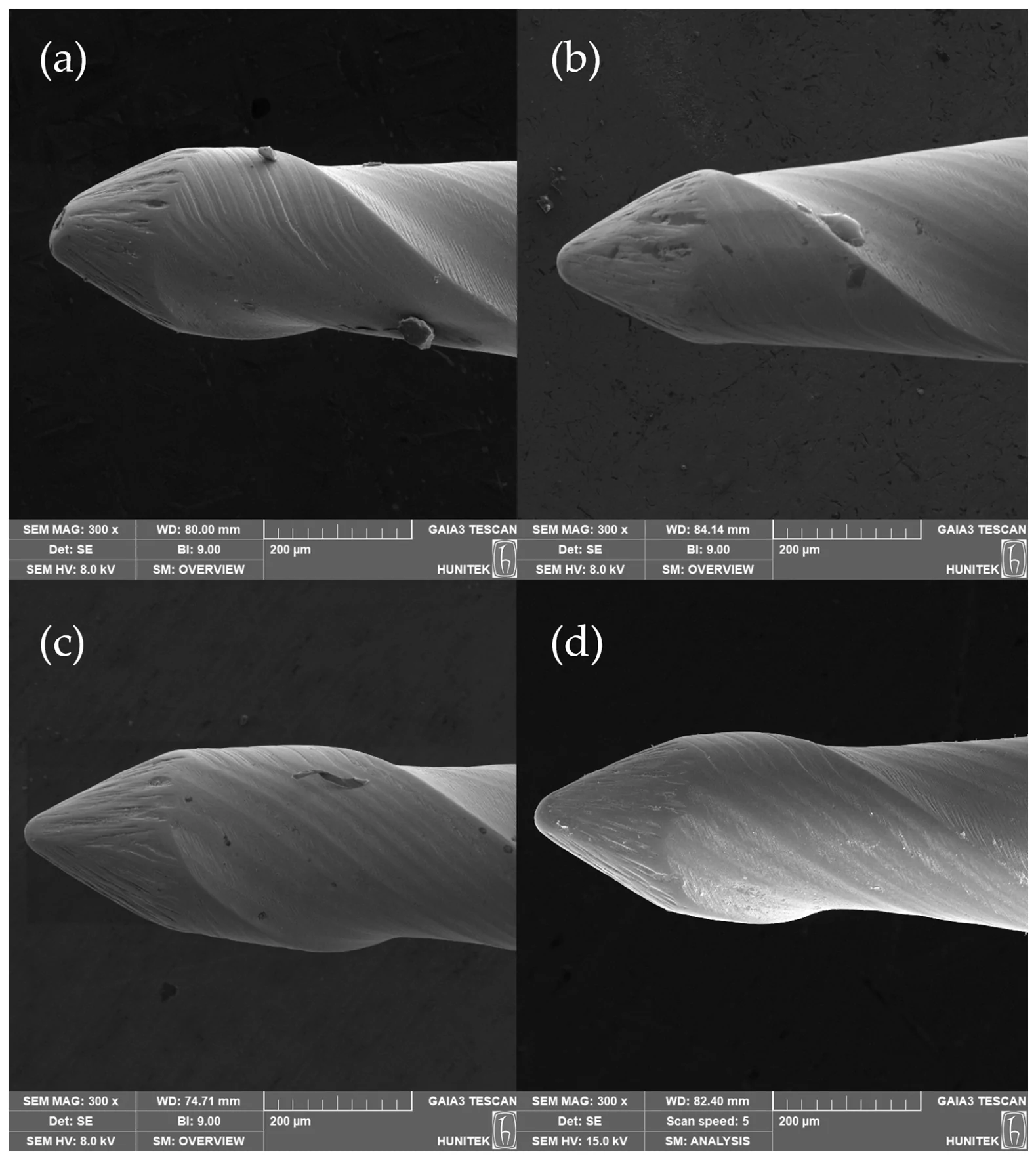
Representative SEM images illustrating the surface features used for morphological evaluation: (**a**) debris, (**b**) corrosion, (**c**) pitting, and (**d**) an unused instrument.

The surface morphology of the instruments was evaluated using SEM. All analyses were performed at a university-based materials characterization laboratory. Following completion of the EDS analyses, the samples were sputter-coated with gold (Au) to improve surface conductivity prior to SEM imaging. SEM examinations were carried out using a GAIA3 TESCAN scanning electron microscope (TESCAN, Brno, Czech Republic). The imaging parameters included the use of a secondary electron (SE) detector, an accelerating voltage of 7.0–15.0 kV, a working distance of 8.0 mm, and a beam intensity of 9.0. A scale bar of 200 µm was used for all images. Each instrument was evaluated at three different regions: apical, middle, and coronal. The magnification levels were selected to provide optimal visualization of the surface features in each region while maintaining an appropriate field of view. Accordingly, the coronal, middle, and apical regions were examined at ×100, ×150, and ×300 magnification, respectively. The following surface characteristics were assessed: debris, corrosion, and pitting formation (Figure 2). For SEM scoring, debris was defined as adherent material covering or partially covering the instrument surface; corrosion was defined as areas showing surface deterioration or irregularity suggestive of chemical degradation; and pitting was defined as discrete, localized depressions or cavities on the instrument surface. Each feature was recorded, using a binary scoring system, as present when identified within the evaluated region and absent when no such feature was observed.

The SEM image evaluations were performed independently by two blinded investigators who were not involved in the image acquisition process. Prior to the analysis, the investigators were calibrated using representative images to standardize the evaluation criteria. Inter-examiner agreement was excellent (Cohen’s κ = 0.91 for debris, 0.88 for corrosion, and 0.86 for pitting). In cases of disagreement, the images were re-evaluated jointly until consensus was reached.

### 2.5. EDS Analysis

The elemental composition of the instrument surfaces was analysed using EDS integrated into the SEM system. The analyses were performed using an Oxford Instruments EDS detector (Oxford Instruments, Oxford, UK). All EDS measurements were performed before gold sputter coating.

The EDS measurements were obtained from predefined regions along the instrument, corresponding to the apical, middle, and coronal segments, based on standardized reference points along the active fluted portion of the file to ensure consistency across all measurements. The elements evaluated included nickel (Ni), titanium (Ti), oxygen (O), carbon (C), phosphorus (P), sodium (Na), chlorine (Cl), and calcium (Ca). For each instrument, three spot measurements were obtained from each of the apical, middle, and coronal regions, resulting in nine EDS measurements per instrument. Measurement points were selected without specifically targeting areas exhibiting corrosion or other visible surface defects. Quantitative spot analyses were performed at ×3000 magnification using an accelerating voltage of 15–20 kV, with a live time of 30–60 s and a dead time of 20–40%. The elemental composition was quantified as weight percentage (wt%).

### 2.6. Statistical Analysis

All statistical analyses were performed using R software (version 4.4.2; R Foundation for Statistical Computing, Vienna, Austria). The level of statistical significance was set at α = 0.05.

SEM data (presence/absence of debris, corrosion, and pitting) were analysed using the Fisher–Freeman–Halton exact test separately for each region. When a significant overall difference was detected, pairwise Fisher’s exact tests were performed between the irrigation protocols within that region. For these three pairwise comparisons, the Bonferroni-adjusted significance threshold was set at *p* < 0.0167 (0.05/3).

Elemental composition data obtained via EDS analysis (Ni, Ti, O, C, P, Na, Cl, and Ca; wt%) were analysed using linear mixed-effects models to account for the hierarchical structure of the data. Given that three measurements (apical, middle, and coronal) were obtained from each instrument, observations were treated as repeated measures within each sample.

The mixed-effect models included the irrigation protocol (DW, NEN, HEDP) as a fixed between-subject factor, and instrument region (apical, middle, coronal) as a fixed within-subject factor, alongside their interaction. To account for intra-sample correlation, a random intercept was incorporated for each instrument. F-tests for fixed effects were calculated using the Satterthwaite approximation. When a significant protocol × region interaction was detected, pairwise comparisons of estimated marginal means were performed between irrigation protocols within each region, with Bonferroni adjustment for multiple comparisons. The data are presented as estimated marginal means ± standard error, and graphical representations were generated using the ggplot2 package.

## 3. Results

### 3.1. SEM Findings

The frequency of debris, corrosion, and pitting observed on the instrument surfaces is presented in Table 1. Debris was observed at varying frequencies across the experimental groups and instrument regions. No overall differences among the groups were detected in the apical (*p* = 1.0000) or coronal (*p* = 0.7537) regions. A significant overall difference was detected in the middle region (*p* = 0.0377); however, none of the pairwise comparisons reached the Bonferroni-adjusted significance threshold.

Regarding corrosion, significant overall differences among the groups were detected in the apical (*p* = 0.0018) and coronal regions (*p* = 0.0006), whereas no significant difference was observed in the middle region (*p* = 0.0658). In the apical region, pairwise comparisons showed a significantly higher corrosion frequency in the NEN group than in the DW group (90% vs. 10%; *p* = 0.0011), while the other pairwise comparisons were not significant. In the coronal region, both the NEN and HEDP groups exhibited significantly higher corrosion frequencies than the DW group (80% and 60% vs. 0%; *p* = 0.0007 and *p* = 0.0108, respectively), whereas no significant difference was found between the NEN and HEDP groups (*p* = 0.6285).

**Figure 3:**
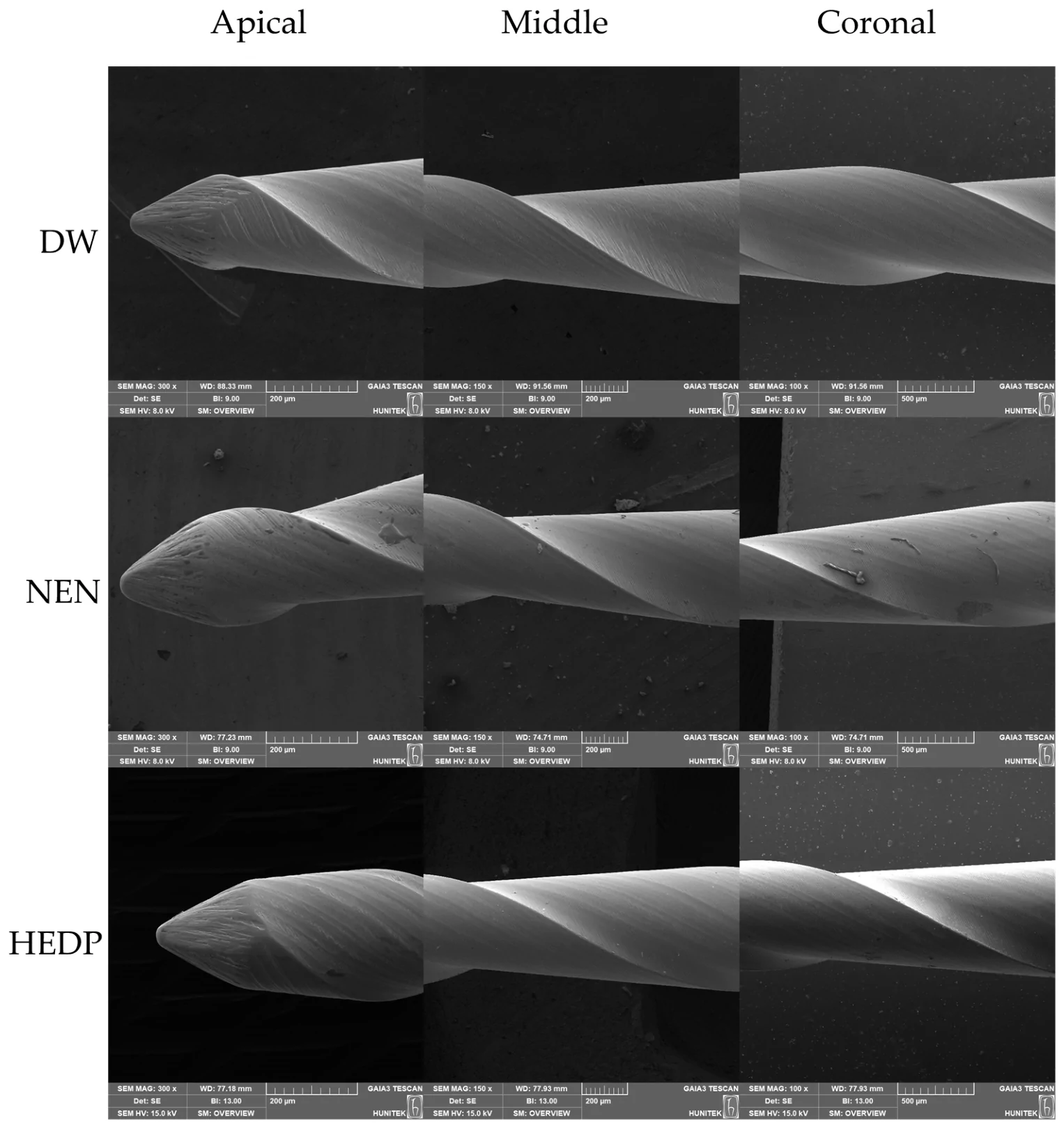
Representative SEM images of the apical, middle, and coronal regions of instruments from the distilled water (DW), sequential NaOCl–EDTA–NaOCl (NEN), and HEDP-based continuous chelation (HEDP) groups.

For pitting, no significant overall difference was observed in the apical region (*p* = 0.2418). Significant overall differences were detected in the middle (*p* = 0.0076) and coronal (*p* = 0.0016) regions. Pairwise comparisons showed significantly higher pitting frequencies in the NEN group than in the DW group in both the middle (60% vs. 0%; *p* = 0.0054) and coronal (70% vs. 0%; *p* = 0.0011) regions. No other pairwise comparisons were statistically significant. Representative SEM images of the experimental groups are shown in Figure 3.

Analysis of the number of affected regions per instrument revealed no significant difference among the groups for debris (*p* = 0.2520) (Table 2). Significant overall differences were observed for corrosion (*p* = 0.0039) and pitting (*p* = 0.0062). Pairwise comparisons showed that the NEN group had a significantly greater number of affected regions than the DW group for both corrosion (*p* = 0.0029) and pitting (*p* = 0.0031). No other pairwise comparisons were statistically significant.

### 3.2. EDS Findings

Linear mixed-effects model analysis demonstrated a significant interaction between irrigation protocol and instrument region for all elements except Cl; detailed model estimates and pairwise comparisons are provided in Appendix A (Table A1 and Table A2).

**Figure 4:**
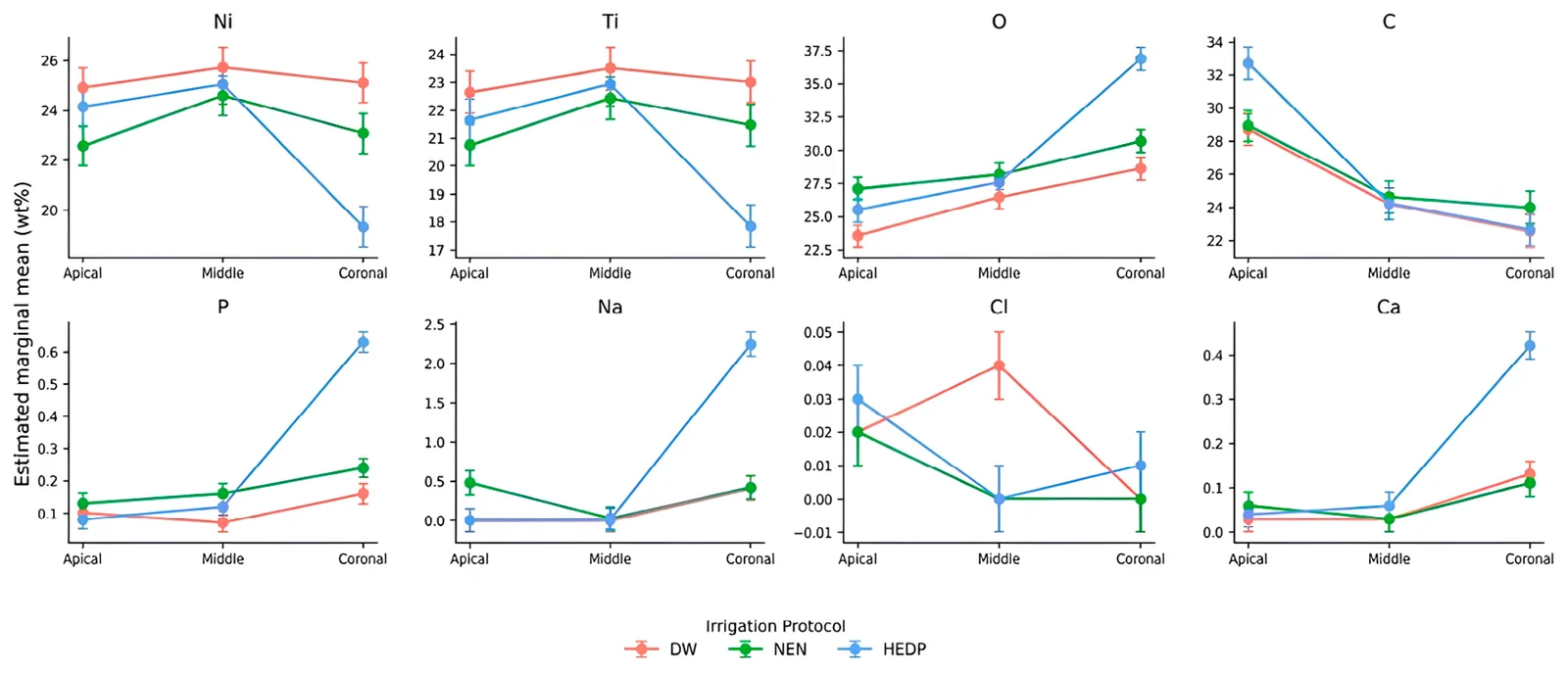
Estimated marginal means of elemental composition (wt%) across instrument regions (apical, middle, and coronal) for each irrigation protocol (DW, NEN, and HEDP). Error bars represent standard errors.

These effects were most pronounced in the coronal region, where the HEDP group exhibited significantly lower Ni and Ti levels, as well as higher O, P, Na, and Ca levels compared to the DW and NEN groups (all *p* < 0.05). In contrast, no consistent inter-group differences were observed in the apical and middle regions. No significant interaction effect was detected for Cl (*p* = 0.058) (Figure 4). 

## 4. Discussion

The present study evaluated the effects of different irrigation protocols on the surface morphology and elemental composition of a heat-treated Ni-Ti single-file instrument. The findings demonstrated protocol- and region-dependent differences in both surface characteristics and elemental composition. SEM analysis showed significantly higher frequencies of corrosion and pitting in the NEN group than in the DW group in selected instrument regions, whereas no significant differences in these outcomes were detected between the NEN and HEDP groups. EDS analysis demonstrated a distinct elemental profile in the HEDP group, particularly in the coronal region, characterized by lower Ni and Ti levels and higher O, P, Na, and Ca levels than in the DW and NEN groups. Therefore, the null hypothesis was rejected.

Sequential use of NaOCl and EDTA is commonly employed in endodontic irrigation because NaOCl provides antimicrobial activity and tissue-dissolving capacity, whereas EDTA is used as a chelating agent for removal of the inorganic component of the smear layer [8,19]. Because EDTA can reduce the available chlorine content and tissue-dissolving capacity of NaOCl when the solutions are combined, they are generally applied separately in sequential irrigation protocols [20].

Continuous chelation with HEDP provides an alternative approach because HEDP can be combined with NaOCl during instrumentation while maintaining chelating activity [2,9]. Accordingly, the present study compared sequential NaOCl–EDTA–NaOCl irrigation with HEDP-based continuous chelation to evaluate their associations with surface morphological and elemental changes in a heat-treated Ni-Ti single-file instrument.

The 20 min exposure period was used as an accelerated experimental condition rather than as a simulation of continuous instrument use in a single root canal. In the present model, the instruments were rotated in the irrigating solutions without contact with dentin or canal walls and were therefore not subjected to the mechanical stresses associated with clinical instrumentation. The extended exposure was intended to isolate protocol-associated chemical surface responses under controlled conditions. Clinically, irrigant exposure may also be cumulative because a single-file instrument can be used sequentially in multiple canals of the same tooth, with irrigation performed repeatedly during preparation [18]. Nevertheless, the 20 min duration should not be regarded as equivalent to a specific clinical instrumentation time, and the magnitude of the observed surface alterations should not be directly extrapolated to routine clinical use. Lin et al. [5] reported little or no apparent surface corrosion after static immersion of several conventional and heat-treated Ni-Ti instruments in 4% NaOCl for 20 min. In contrast, the instruments in the present study were continuously rotated at 400 rpm throughout the 20 min exposure and were subjected to different irrigation protocols rather than NaOCl alone. In addition, differences in instrument metallurgy, manufacturing procedures, and surface characteristics may contribute to differences in corrosion susceptibility among Ni-Ti systems [5]. Therefore, direct comparison of the magnitude of surface alterations between the two experimental models should be made with caution.

The influence of chelating agents on corrosion-related surface changes has been investigated previously [21]. Samara et al. [6] reported less corrosion following sequential EDTA and NaOCl exposure than with NaOCl alone. In the present study, however, the NEN protocol was associated with significantly higher corrosion frequencies than DW in the apical and coronal regions and with significantly higher pitting frequencies in the middle and coronal regions. The HEDP group also exhibited significantly higher corrosion than DW in the coronal region, whereas no significant differences in corrosion or pitting were detected between the NEN and HEDP groups. These differences from previous reports may reflect variations in the tested Ni-Ti systems, exposure conditions, irrigant concentrations, and irrigation protocols [6]. Because a NaOCl-only group was not included, the individual contribution of NaOCl and the potential modifying effects of the chelating agents cannot be determined from the present experimental design.

Importantly, the morphological and elemental findings were not fully concordant. Previous studies combining SEM and EDS have also shown that these methods provide complementary information on the morphological and elemental characteristics of Ni-Ti instrument surfaces [22]. This discordance suggests that morphological deterioration and compositional modification of the instrument surface may represent distinct responses to irrigant exposure. One possible explanation is modification of the passive oxide-rich surface layer of the Ni-Ti alloy. The lower relative Ni and Ti levels accompanied by higher O levels observed in the HEDP group may be compatible with changes in the composition or coverage of this surface layer, although EDS cannot determine its structure or thickness. Elemental dissolution may also contribute to such compositional changes, as previous studies have demonstrated the release of alloy constituents from Ni-Ti instruments following exposure to endodontic irrigants [6]. In addition, exposure to etidronic acid has been reported to alter the surface topography of Ni-Ti instruments, supporting the possibility of a direct interaction between HEDP-containing solutions and the instrument surface [23]. Changes in crystalline phase represent another possible contributor to the response of heat-treated Ni-Ti instruments because their phase constitution may include varying proportions of austenite, martensite, and R-phase depending on thermomechanical processing [24,25]. However, no phase-specific analysis was performed in the present study; therefore, whether the tested irrigation protocols affected the crystalline phase cannot be determined. Likewise, EDS cannot distinguish intrinsic changes in the alloy or oxide layer from superficial deposits or determine the chemical state of the detected elements. These mechanisms should therefore be regarded as plausible interpretations rather than demonstrated surface reactions.

The EDS findings further demonstrated that protocol-associated differences in elemental composition were region-dependent and were most evident in the coronal region. Significant protocol × region interactions were detected for Ni, Ti, O, C, P, Na, and Ca, whereas the interaction for Cl did not reach statistical significance. Regarding C, carbon detected by EDS on metallic surfaces may reflect surface contamination, and variations in surface C levels have also been reported in Ni-Ti endodontic instruments following exposure to different irrigants [6]. The Na and Cl findings should be interpreted cautiously because saline (0.9% NaCl) was used as an intermediate rinse in the NEN protocol and as a final rinse in all groups. Saline has also been used as an exposure medium in previous investigations of the electrochemical behaviour of Ni-Ti endodontic instruments [12,26]. Therefore, the contribution of the saline rinsing steps to the detected surface Na and Cl cannot be excluded. In the coronal region, the HEDP group exhibited lower Ni and Ti and higher O, P, Na, and Ca levels than the DW and NEN groups. The higher Ca level in the HEDP group is noteworthy because the experimental model did not include dentin or another calcium-containing substrate; therefore, it cannot be attributed to dentin-derived Ca^2+^. Moreover, EDS identifies elemental composition but does not establish the source or chemical state of the detected elements. Previous surface analyses have also detected Ca and other non-alloy elements on untreated Ni-Ti instruments and attributed their presence to environmental contamination [13]. Therefore, the Ca detected in the present study cannot be conclusively attributed to the irrigation protocol.

An important aspect of the present findings was the region-dependent response of the instrument surface. The predominance of elemental differences in the coronal region indicates that the surface response was not uniform along the working portion of the instrument. Instrument geometry varies along the working portion of Ni-Ti instruments, while manufacturing procedures may introduce surface irregularities and microstructural defects that influence their surface characteristics [6,27]. These factors may contribute to a heterogeneous response along the instrument. Because the present EDS measurements were obtained from predefined apical, middle, and coronal regions under otherwise standardized exposure conditions, the regional pattern is unlikely to reflect differences in exposure duration alone. Nevertheless, the relative contributions of instrument geometry and pre-existing surface characteristics cannot be distinguished by the present design.

Although exposure to endodontic irrigants has been shown to affect the mechanical properties of Ni-Ti instruments in previous studies [28,29,30], the present study evaluated only morphological and elemental surface characteristics. No mechanical testing, such as cyclic fatigue or fracture resistance assessment, was performed. Therefore, the observed surface changes should not be interpreted as direct evidence of altered mechanical or clinical performance. The mechanical and clinical implications of these findings remain uncertain and require further investigation.

The findings of the present study should be interpreted within the limitations of its experimental design. First, the standardized immersion model does not fully reproduce the mechanical, thermal, and biological conditions encountered during clinical root canal instrumentation. The absence of dentin and other canal-derived substrates may also have influenced the observed surface elemental profiles. In addition, all experiments were conducted at room temperature, and the effects of temperature-dependent changes in irrigant reactivity were not evaluated. Second, a NaOCl-only group was not included because the study was designed to compare combined irrigation protocols rather than the effects of individual solutions; therefore, the contribution of individual irrigant components to the observed surface changes cannot be determined. Third, only one heat-treated Ni-Ti instrument system was evaluated, and the findings should therefore not be generalized to instruments with different alloys, heat treatments, manufacturing procedures, or surface characteristics. Fourth, unused instruments were not subjected to EDS analysis; therefore, the baseline elemental composition of the tested instrument system could not be directly characterized. Quantitative EDS comparisons were based on point analyses, whereas elemental mapping was used only for representative qualitative visualization. Finally, SEM outcomes were assessed using a binary presence/absence approach rather than a graded severity scale. Although this approach provided reproducible classification, as reflected by the high inter-observer agreement, it does not capture differences in the extent or severity of surface alterations. Future studies should evaluate different Ni-Ti systems under clinically simulated instrumentation conditions and incorporate mechanical testing and graded or quantitative approaches to surface characterization.

## 5. Conclusions

Under the present experimental conditions, the tested irrigation protocols were associated with region-dependent morphological and elemental differences in Super One File instruments. Compared with DW, the NEN protocol was associated with significantly higher frequencies of corrosion and pitting in selected instrument regions, whereas no significant differences in these outcomes were detected between the NEN and HEDP protocols. The HEDP-based protocol exhibited a distinct elemental profile, particularly in the coronal region, despite the absence of proportionate morphological deterioration. These findings are specific to the tested instrument and experimental exposure conditions and should not be interpreted as direct evidence of altered mechanical or clinical performance.

## Figures and Tables

**Table 1:** Frequency of debris, corrosion, and pitting across groups and regions.

	Debris			Corrosion			Pitting		
	Apical	Middle	Coronal	Apical	Middle	Coronal	Apical	Middle	Coronal
	n (%)	n (%)	n (%)	n (%)	n (%)	n (%)	n (%)	n (%)	n (%)
DW	10 (100%)	6 (60%)	8 (80%)	1 (10%) ^a^	1 (10%)	0 (0%) ^a^	1 (10%)	0 (0%) ^a^	0 (0%) ^a^
NEN	10 (100%)	10 (100%)	10 (100%)	9 (90%) ^b^	6 (60%)	8 (80%) ^b^	4 (40%)	6 (60%) ^b^	7 (70%) ^b^
HEDP	10 (100%)	5 (50%)	9 (90%)	6 (60%) ^ab^	5 (50%)	6 (60%) ^b^	1 (10%)	2 (20%) ^ab^	2 (20%) ^ab^
*p*	1.0000	0.0377	0.7537	0.0018	0.0658	0.0006	0.2418	0.0076	0.0016

Data are presented as n (%). *p* values were obtained using the Fisher–Freeman–Halton exact test. Different superscript letters within a row indicate significant pairwise differences (Fisher’s exact test, Bonferroni-adjusted *p* < 0.0167). No pairwise differences were significant for middle-region debris.

**Table 2:** Number of affected regions per instrument.

Surface Feature	Group	0 Regions	1 Region	2 Regions	3 Regions	*p*
Debris	DW	0	2	2	6	0.2520
	NEN	0	0	0	10	
	HEDP	0	1	4	5	
Corrosion	DW ^a^	8	2	0	0	0.0039
	NEN ^b^	1	1	2	6	
	HEDP ^ab^	2	2	3	3	
Pitting	DW ^a^	9	1	0	0	0.0062
	NEN ^b^	1	4	2	3	
	HEDP ^ab^	6	3	1	0	

*p* values were obtained using the Fisher–Freeman–Halton exact test. Different superscript letters within each surface feature indicate significant pairwise differences (Fisher’s exact test, Bonferroni-adjusted *p* < 0.0167).

## Data Availability

The underlying quantitative data supporting the findings of this study, including the EDS measurements and SEM scoring data, are provided in the Supplementary Materials. Further inquiries can be directed to the corresponding author.

## Supplementary Materials

The following supporting information can be downloaded at: https://www.mdpi.com/article/10.3390/bioengineering13091061/s1, Table S1: Database; Table S2: DW; Table S3: NEN; Table S4: HEDP.

## Abbreviations

The following abbreviations are used in this manuscript:

Ni-Ti	Nickel-Titanium
SEM	Scanning electron microscopy
EDS	Energy-dispersive X-ray spectroscopy
NaOCl	Sodium hypochlorite
EDTA	Ethylenediaminetetraacetic acid
HEDP	1-hydroxyethylidene-1,1-diphosphonic acid

## Appendix A

**Table A1:** Regional post hoc comparisons.

Element	Region	Comparison	*p*-Value
Ni	Coronal	DW vs. HEDP	<0.001
Ni	Coronal	NEN vs. HEDP	0.004
Ti	Coronal	DW vs. HEDP	<0.001
Ti	Coronal	NEN vs. HEDP	0.003
O	Apical	DW vs. NEN	0.016
O	Coronal	DW vs. HEDP	<0.001
O	Coronal	NEN vs. HEDP	<0.001
C	Apical	DW vs. HEDP	0.014
C	Apical	NEN vs. HEDP	0.023
P	Coronal	DW vs. HEDP	<0.001
P	Coronal	NEN vs. HEDP	<0.001
Na	Coronal	DW vs. HEDP	<0.001
Na	Coronal	NEN vs. HEDP	<0.001
Ca	Coronal	DW vs. HEDP	<0.001
Ca	Coronal	NEN vs. HEDP	<0.001

Estimated marginal means were obtained from the linear mixed-effects models and represent model-adjusted values accounting for within-instrument correlation. Pairwise comparisons were performed with Bonferroni correction.

**Table A2:** Model-adjusted elemental composition (wt%).

Element	Region	DW (Mean ± SE)	NEN (Mean ± SE)	HEDP (Mean ± SE)	F (df)	*p*-Value
Ni	Apical	24.92 ± 0.81	22.57 ± 0.81	24.15 ± 0.81	F(4,54.83) = 4.89	0.002
	Middle	25.73 ± 0.81	24.60 ± 0.81	25.05 ± 0.81		
	Coronal	25.11 ± 0.81	23.08 ± 0.81	19.30 ± 0.81		
Ti	Apical	22.64 ± 0.75	20.74 ± 0.75	21.65 ± 0.75	F(4,54.91) = 4.31	0.004
	Middle	23.50 ± 0.75	22.43 ± 0.75	22.94 ± 0.75		
	Coronal	23.01 ± 0.75	21.47 ± 0.75	17.84 ± 0.75		
O	Apical	23.57 ± 0.87	27.10 ± 0.87	25.51 ± 0.87	F(4,54.91) = 8.32	<0.001
	Middle	26.45 ± 0.87	28.16 ± 0.87	27.59 ± 0.87		
	Coronal	28.61 ± 0.87	30.66 ± 0.87	36.88 ± 0.87		
C	Apical	28.71 ± 0.97	28.93 ± 0.97	32.70 ± 0.97	F(4,54.69) = 2.95	0.028
	Middle	24.19 ± 0.97	24.62 ± 0.97	24.24 ± 0.97		
	Coronal	22.57 ± 0.97	23.99 ± 0.97	22.66 ± 0.97		
P	Apical	0.10 ± 0.03	0.13 ± 0.03	0.08 ± 0.03	F(4,55.03) = 26.21	<0.001
	Middle	0.07 ± 0.03	0.16 ± 0.03	0.12 ± 0.03		
	Coronal	0.16 ± 0.03	0.24 ± 0.03	0.63 ± 0.03		
Na	Apical	0.00 ± 0.15	0.48 ± 0.15	0.01 ± 0.15	F(4,81) = 19.58	<0.001
	Middle	0.00 ± 0.15	0.03 ± 0.15	0.01 ± 0.15		
	Coronal	0.41 ± 0.15	0.42 ± 0.15	2.25 ± 0.15		
Cl	Apical	0.02 ± 0.01	0.02 ± 0.01	0.03 ± 0.01	F(4,54.61) = 2.43	0.058
	Middle	0.04 ± 0.01	0.00 ± 0.01	0.00 ± 0.01		
	Coronal	0.00 ± 0.01	0.00 ± 0.01	0.01 ± 0.01		
Ca	Apical	0.03 ± 0.03	0.06 ± 0.03	0.04 ± 0.03	F(4,54.89) = 9.66	<0.001
	Middle	0.03 ± 0.03	0.03 ± 0.03	0.06 ± 0.03		
	Coronal	0.13 ± 0.03	0.11 ± 0.03	0.42 ± 0.03		

Values are presented as estimated marginal means ± standard error. F and *p* values correspond to the protocol × region interaction in the linear mixed-effects models.
